# Body balance in elderly patients, 12 months after treatment for BPPV

**DOI:** 10.5935/1808-8694.20130008

**Published:** 2015-10-14

**Authors:** Solange Martiliano Lança, Juliana Maria Gazzola, Cristiane Akemi Kasse, Fatima Cristina Alves Branco-Barreiro, Daniela Patricia Vaz, Renata Coelho Scharlach

**Affiliations:** Speech and Hearing Therapist. Audiologist. MSc in Body Balance Rehabilitation and Social Inclusion; Head of the Department of Technical Projects and Campaigns of the Secretary of the Rights of People with Disabilities of the City of Barueri - SP; Gerontologist - UNIFESP and SBGG. MSc in Sciences - Federal University of São Paulo (UNIFESP). Professor and Researcher - MSc Program in Body Balance Rehabilitation and Social Inclusion - Bandeirante University - São Paulo - Brazil; PhD in Sciences - UNIFESP. Professor and Researcher - MSc Program in Body Balance Rehabilitation and Social Inclusion - UNIBAN; Speech and Hearing Therapist - PhD in Neurosciences and Behavior - USP. Professor and Researcher - MSc Program in Body Balance Rehabilitation and Social Inclusion - UNIBAN; MSc in Body Balance Rehabilitation and Social Inclusion. Professor and Coordinator - Método School - São Paulo; Speech and Hearing Therapist. PhD in Sciences - UNIFESP. Professor and Researcher - MSc Program in Body Balance Rehabilitation and Social Inclusion - UNIBAN. Bandeirante University - São Paulo - Brazil

**Keywords:** aged, dizziness, postural balance, rehabilitation, vestibular diseases

## Abstract

Benign Paroxysmal Positional Vertigo is highly prevalent in the elderly population, triggering major changes in body balance.

**Objective:**

To compare the results obtained from static posturography in the elderly before and after otoliths repositioning maneuvers and 12 months after treatment onset. Design: longitudinal, descriptive and analytical study.

**Method:**

Elderly patients with clinical diagnosis of BPPV submitted to Balance Rehabilitation Unit static posturography in 10 sensory conditions at three time intervals: before and after the repositioning maneuver and12 months after the treatment.

**Results:**

We studied 23 subjects with a mean age of 68.74 years. Posturography revealed that the stability limit was not significantly different when the three time intervals were compared (*p* = 0.405). The center of pressure (CoP) showed a significant change in condition 2 (stable surface and closed eyes), because after the repositioning maneuver, the CoP significantly differed vis-à-vis the results before and 12 months after the treatment (*p* = 0.003). The values of body velocity sway (BVS) were significantly different in six sensory conditions in these three time intervals.

**Conclusion:**

12 months after the treatment for BPPV, the static posturography showed balance abnormalities similar to those found before treatment.

## INTRODUCTION

Progress and the expansion of services in the fields of social works, healthcare, leisure and occupational therapy has brought about a very positive impact to the aspects involving the longevity of the world population. With this, and according to data from the Brazilian Institute of Geography and Statistics - IBGE – 2000 census, the number of people with 60 years of age or more has been increasing significantly in Brazil, with projections saying that by 2020 or 2025, the number of elderly citizens will be over 30 million^1^.

Benign Positional Paroxysmal Vertigo (BPPV) is one of the most prevalent neurotology disorder in the elderly^2^ and it may bring about changes to body balance which directly impact the quality of life of these individuals. This disease has a clinical setting characterized by sudden vertigo fits, usually intense, lasting for seconds, triggered by certain movements of the head^3^.

BPPV's main etiology is idiopathic, followed by head injuries, general anesthesia, *diabetes mellitus*, hypothyroi-dism, Ménière's disease, vestibular neuritis, viral labyrinthitis, posterior fossa tumors and ischemia, and others^4^.

BPPV's diagnosis is achieved by analyzing symptoms and vertigo or positional nystagmus triggered by the Dix-Hallpike maneuver, the Side Lying Maneuver or Head Roll Maneuver^4^^,^^5^.

Treatment is based on repositioning the statoconia (in maneuvers such as Epley, Lempert and Gufoni), freeing maneuvers (Semont) or habituation, such as the Brandt-Daroff^6^ maneuver. These may or may not be associated with the use of medication, depending on disease etiology, associated comorbidities and possible sequelae (e.g.: phobic vertigo).

In recent years, some virtual reality systems and dynamic and static force platforms were developed, aiming at improving body balance assessment and rehabilitation methods for our balance systems^7^. Among them, we stress the Balance Rehabilitation Unit (BRU^TM^). This equipment assesses balance disorders by measuring pressure center (PC) shifting areas and body sway velocity (BSV) under ten sensory situations associated with vestibular, visual and somatosensory reflexes. The current literature^7^^,^^8^, has studies involving the use of virtual reality to assess patients with BPPV and other diseases, but we did not find any study using such technology to follow these patients after discharge.

Body balance assessment and rehabilitation in the elderly have been receiving a lot a attention recently, favoring a diagnosis of BPPV, and the importance of the differential diagnosis for the early treatment of labyrinthine diseases.

This study aims at comparing the results obtained from static posturography in elderly patients before and after the otoliths repositioning maneuvers (ORM) and after 12 months of initial treatment for BPPV.

## METHOD

This is a longitudinal, descriptive and analytical study, carried out in the Laboratory of Studies and Research of a Professional Master's Degree Program in São Paulo, from August 2010 through May of 2011. The study was approved by the Ethics and Norms Committee of the University, under protocol number 115/10.

Before starting the study, all the patients who accepted to participate were instructed about it by means of an information letter, and its data was used only after the patients signed the Informed Consent Form.

Of the 45 patients who made up the sample in this study, 21 were volunteers, with ages ranging between 60 and 70 years, mean age of 68.74 years, from both genders, 82.6% were females and 17.4% were males.

The following inclusion and exclusion criteria were established: a) Inclusion criteria: male and female volunteers, with ages equal to or higher than 60 years, from the laboratory of studies and research; patients submitted to BPPV treatment by means of ORM for the past 12 months, being medically discharged; patients with or without a new vertigo episode with BPPV characteristics within 12 months after medical discharge; b) Exclusion criteria: patients unable to understand and follow a simple verbal command; patients unable to remain standing up by themselves; patients with severe visual impairment, or not compensated by corrective lenses; patients with orthopedic disorders resulting in movement limitations or use of lower limb prosthesis; patients with neurological and/or psychiatric disorders; patients reporting alcohol drinking 24 hours prior to the assessment; patients using medication acting on the Central Nervous System or Vestibular System; patients submitted to body balance rehabilitation after medical discharge.

In order to better characterize the population, we interviewed the patients, stressing a past of falls, dizziness, dizziness recurrence and the use of medication in the past 12 months. For the static posturography, we utilized the Balance Rehabilitation Unit (BRU^TM^). This unit has a posturography mode integrated with visual stimuli, which are projected by a pair of virtual reality goggles, used to assess patients with balance disorders, vertigo or instability^9^. Moreover, it has a rehabilitation module and one of posture training games.

This equipment includes a computer with the BRU^TM^ software, safety structure (metal struts and harness), force platform, virtual reality goggles, accelerometer and foam pillow. Static posturography was carried out in a silent room, with reduced lighting in environment conditions favoring assessment, preventing external factors from impacting the tests^10^.

**Figure 1:**
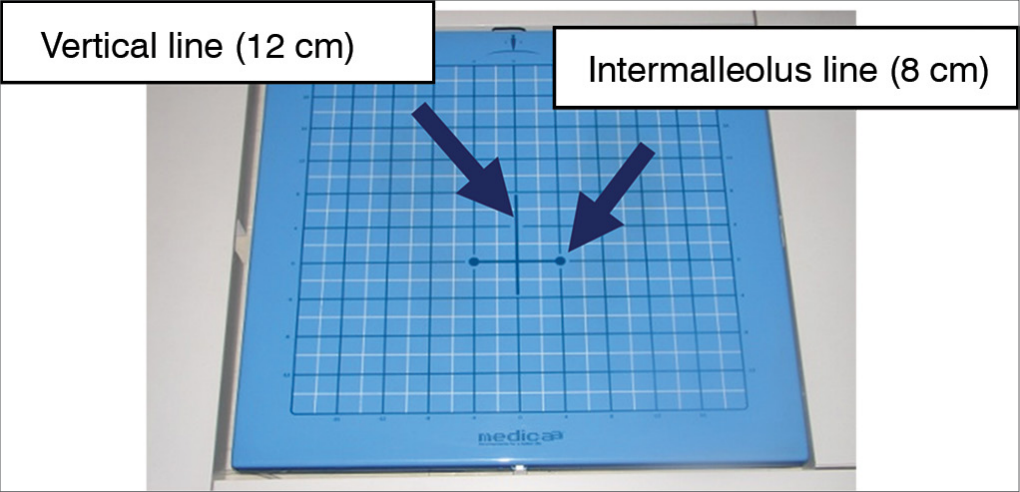
BRU^TM^ Platform. Personal files.

BRU^TM^ Force Platform, has an area of 40 cm × 40 cm, including vertical and horizontal coordinates; it has an 8 cm horizontal (side-to-side) line (intermalleolus line) to position the patient's feet and a 12 cm vertical line to intercept the middle point of the intermalleolus line^7^. The platform has four load-cell-type force sensors, which are distributed to measure the three force components and the three force movement components (anteroposterior, mediolateral and vertical directions) (Figure 1).

This platform converts the pressure applied on it into electrical signs, enabling to establish information about the patient's pressure center position by means of quantitative indicators: stability limit (SL), PC area (cm^2^) and BSV (cm/s) under ten sensory situations^9^.

In order to establish the SL, the patients were properly positioned in the platform and were instructed to shift they bodies in the anteroposterior and side-to-side directions, by means of utilizing the ankle, without moving the feet or employing the trunk or hip. The patients moved slowly until reaching their body stability limit, that is, until they felt a little unstable. The movements were carried out in the following sequence: forward; back to the initial position; to the right; back to the initial position; to the left; back to the initial position; backwards; back to the initial position. The patients were asked to perform this task twice, without necessarily completing the 60 seconds saved for this procedure.

To assess the PC and the BSV areas, under the ten sensory situations, the patients were then instructed to maintain a calm standing position, arms extended along the body, for 60 seconds in the following positions:

Situation 1 - standing up on a firm surface with eyes open;
Situation 2 - standing up on a firm surface with eyes closed;
Situation 3 - standing up on a foam pillow surface with eyes open;
Situation 4 - standing up on a firm surface, with saccadic stimulation;
Situation 5 - standing up on a firm surface, with optokinetic stimulation in the horizontal direction, from left to right;
Situation 6 - standing up on a firm surface, with optokinetic stimulation in the horizontal direction, from right to left;
Situation 7 - standing up on a firm surface, with optokinetic stimulation in the vertical direction from the top down;
Situation 8 - standing up on a firm surface, with optokinetic stimulation in the vertical direction from bottom up;
Situation 9 - standing up on a firm surface, with optokinetic stimulation on the horizontal direction, associated to slow and uniform rotation movements of the head;
Situation 10 - standing up on a firm surface, with optokinetic stimulation in the vertical direction, associated with slow and uniform movements of head extension and flexion.

The virtual reality goggles were utilized in the assessments of the fourth to the tenth situation in order to present the saccadic and optokinetic stimuli and vestibule-visual interaction. Eye glasses were allowed, when necessary.

**Figure 2:**
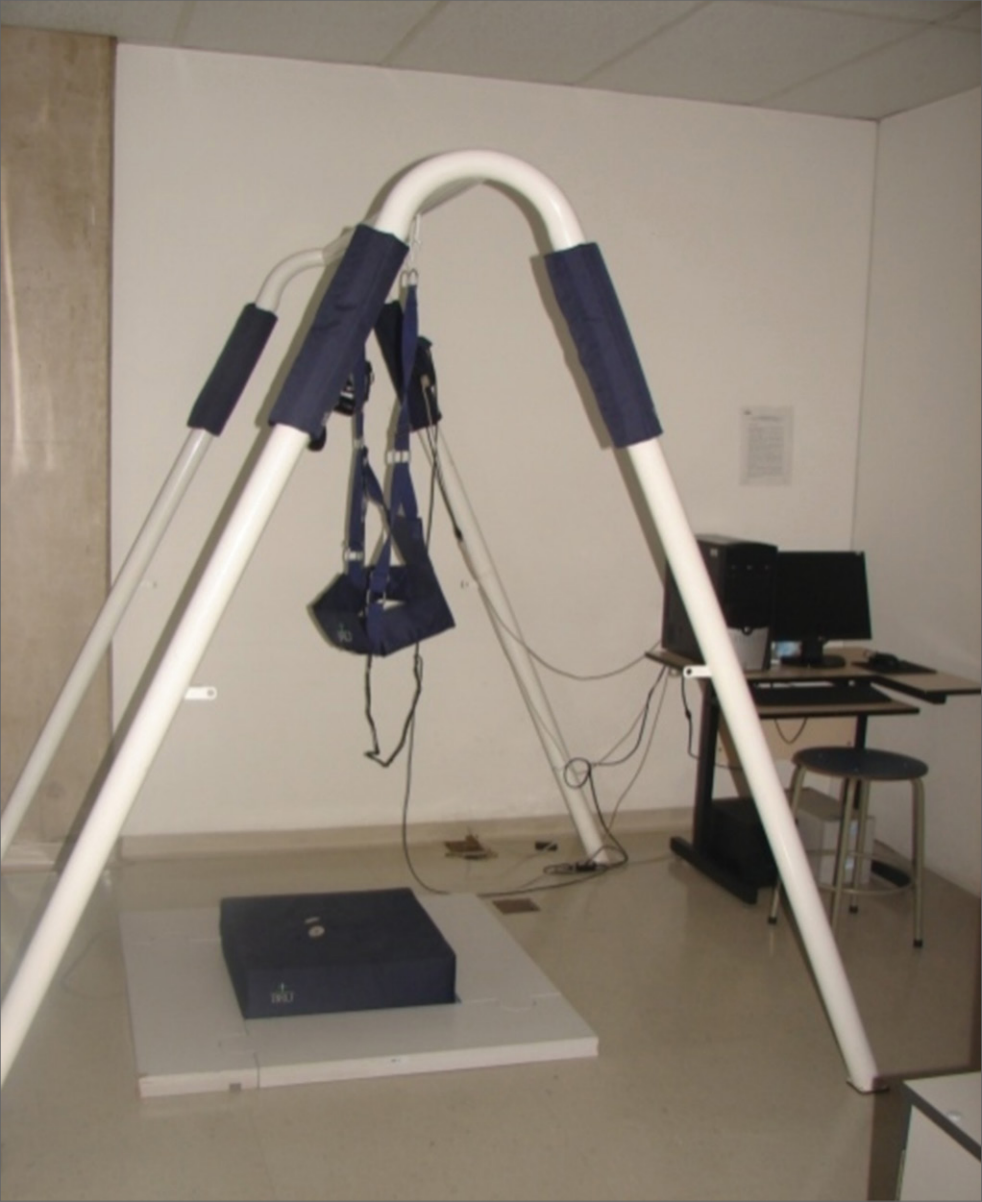
Static posturography - BRU^TM^. Personal files.

During the procedures, the patient was allowed rest times, according to need. Patient safety, concerning the risk of an eventual fall during the assessment, was guaranteed by the presence of an examiner near the patient, and the use of protection equipment (Figure 2).

Initially, we carried out a simple descriptive analysis to characterize the sample. In order to compare the groups along the assessments, we used the non-parametric Friedman test and the significance level was 5%.

## RESULTS

We analyzed the SL obtained at the three assessment moments. The results obtained from the statistical analysis may be seen on Table 1. The sample was made up by 21 individuals in this assessment, because two of them did not have, in their charts, the analyses regarding the SL after treatment (VR).

**Table 1:** Descriptive values and comparative analysis of the SL (cm^2^) before, after and 12 months after treatment of elderly patients with Benign Positional Paroxysmal Vertigo (n = 21).

SL	n	Mean	SD	Median	Minimum	Maximum	*p*
Before maneuver	21	148.86	64.07	140	22	279	
After the maneuver	21	169.14	56.31	174	57	261	0.405
After 1 year	21	151.71	59.02	155	50	246	

Friedman's non-parametric test. SL: Stability Limit. Source: personal files.

The SL did not present significant difference (*p* = 0.405) when the three moments were compared: before (148.86 ± 64.07), after (169.14 ± 56.31) treatment, and after 12 months (151.71 ± 59.02).

Table 2 shows the descriptive value and the comparative analysis of the pressure center (PC) of each one of the situations on the static posturography module in the Balance Rehabilitation Unit (BRU™) before, after treatment and 12 months after treatment.

**Table 2:** Descriptive values and comparative analysis of the PC area (cm^2^) before and after the maneuver, and 12 months after treatment for BPPV in elderly patients (n = 21).

Situations BRU^TM^	Moment	Mean (SD)	Median	Variation	*p*	Moment	*p*
	Before	3.31 (2.87)	2.28	0.81-10.95		Before-After	
Situation 1	After	5.06 (7.52)	2.53	0.48-29.66	0.651	Before-After 1 year	> 0.05 ns
	After 1 year	3.53 (3.67)	2.34	0.94-16.69		After-After 1 year	
	Before	4.84 (3.87)	4.71	0.57-13.32		Before-After	< 0.05*
Situation 2	After	3.24 (3.81)	2.11	0.30-15.13	0.003*	Before-After 1 year	> 0.05 ns
	After 1 year	5.41 (7.17)	3.83	0.91-28.41		After-After 1 year	< 0.05*
	Before	11.79 (9.55)	8.08	2.59-43.77		Before-After	
Situation 3	After	8.98 (10.14)	5.16	1.98-49.46	0.129	Before-After 1 year	> 0.05 ns
	After 1 year	10.29 (7.11)	7.24 1.86	2.01-27.46		After-After 1 year	
	Before	2.35 (2.03)	1.86	0.53-9.48		Before-After	
Situation 4	After	2.42 (2.33)	1.56	0.41-11.37	0.264	Before-After 1 year	> 0.05 ns
	After 1 year	3.70 (4.72)	2.31	0.67-22.60		After-After 1 year	
	Before	2.46 (2.55)	1.79	0.41-12.44		Before-After	
Situation 5	After	2.88 (3.37)	1.52	0.56-13.56	0.172	Before-After 1 year	> 0.05 ns
	After 1 year	3.71 (5.99)	2.25	0.53-28.71		After-After 1 year	
	Before	1.91 (1.57)	1.44	0.42-7.83		Before-After	
Situation 6	After	3.04 (3.82)	1.65	0.40-14.62	0.129	Before-After 1 year	> 0.05 ns
	After 1 year	4.43 (5.85)	2.86	0.75-24.53		After-After 1 year	
	Before	2.28 (1.87)	1.80	0.57-8.58		Before-After	
Situation 7	After	2.78 (3.83)	1.92	0.57-16.84	0.097	Before-After 1 year	> 0.05 ns
	After 1 year	4.50 (5.04)	2.33	0.51-18.84		After-After 1 year	
	Before	2.85 (2.85)	2.02	0.30-12.30		Before-After	> 0.05 ns
Situation 8	After	2.89 (4.45)	1.47	0.23-20.95	0.009*	Before-After 1 year	< 0.05*
	After 1 year	4.37 (4.77)	2.19	0.72-21.21		After-After 1 year	< 0.05*
	Before	5.02 (4.52)	3.70	0.59-21.52		Before-After	> 0.05 ns
Situation 9	After	4.79 (5.79)	3.12	1.34-27.90	0.002*	Before-After 1 year	< 0.05*
	After 1 year	7.75 (6.21)	5.04	1.37-29.04		After-After 1 year	< 0.05*
	Before	4.62 (4.19)	3.24	1.12-20.76		Before-After	
Situation 10	After	4.01 (3.35)	3.27	0.94-15.64	< 0.001*	Before-After 1 year	< 0.05*
	After 1 year	7.51 (7.60)	5.45	1.73-37.99		After-After 1 year	

Friedman's non-parametric test. PC cm^2^ pressure center; Before: before the maneuver; After: after the maneuver; 12 months after the treatment; Situation 1: firm surface and open eyes; Situation 2: firm surface and closed eyes; Situation 3: pillow and eyes closed: Situation 4: firm surface and saccadic stimulation; Situation 5: firm surface and horizontal left/right optokinetic stimulation; Situation 6: firm surface and right/left horizontal optokinetic stimulation; Situation 7: firm surface and vertical top/bottom optokinetic stimulation; Situation 8: firm surface and bottom/up vertical optokinetic stimulation; Situation 9: firm surface and horizontal optokinetic stimulation associated with head movement; Situation 10: firm surface and vertical optokinetic stimulation associated with head movement; ns: not-significant.

In situations 1 (*p* = 0.651), 3 (*p* = 0.129), 4 (*p* = 0.264), 5 *(p=* 0.172), 6 *(p=* 0.129) and 7 *(p=* 0.097) there was no significant difference between the values found in the three moments of assessment.

In situation 2 (*p* = 0.003), There was a significant alteration, because in the after moment, the PC area (3-24 cm^2^) was significantly different when comparing before (4.84 cm^2^) and 12 months after treatment (5.41 cm^2^; *p <* 0.05) and before treatment was not different from the assessment 12 months after treatment *(p>* 0.05). the analysis also showed a significant difference in situations 8 (*p* = 0.009), 9 *(p=* 0.002) and 10 *(p<* 0.001), in which 12 months after treatment, the PC area was different before *(p<* 0.05) and after *(p <* 0.05). The before moment was not different in situations 8 and *9 (p>* 0.05), differing only in situation 10 *(p<* 0.05), that is, under these conditions, the PC area value was significantly higher after 12 months of treatment.

Table 3 shows the descriptive value and the comparative analysis of the results found upon BSV before, after treatment and after 12 months of treatment for BPPV, of the patients who made up the sample in the 10 posturography sensory situations.

**Table 3:** Descriptive values and comparative analysis of the BSV (cm/s) before and after the maneuver, and 12 months after treatment in elderly with Benign Positional Paroxysmal Vertigo (n = 21).

Situations BRU™	Moment	Mean (SD)	Median	Variation	*p*	Moment	*p*
	Before	0.96 (0.36)	0.86	0.57-2.01		Before-After	< 0.05*
Situation 1	After	0.86 (0.34)	0.74	0.48-1.99	0.044*	Before-After 1 year	> 0.05 ns
	After 1 year	0.96 (0.41)	0.85	0.57-2.47		After-After 1 year	< 0.05*
	Before	1.35 (0.61)	1.22	0.52-2.90		Before-After	< 0.05*
Situation 2	After	1.06 (0.52)	0.90	0.42-2.69	0.002*	Before-After 1 year	> 0.05 ns
	After 1 year	1.39 (0.66)	1.17	0.84-3.26		After-After 1 year	< 0.05*
	Before	2.46 (1.01)	2.13	1.08-4.60		Before-After	< 0.05*
Situation 3	After	2.02 (0.87)	1.80	1.00-4.65	0.001*	Before-After 1 year	> 0.05 ns
	After 1 year	2.55 (1.07)	2.24	0.97-5.03		After-After 1 year	< 0.05*
	Before	1.25 (0.43)	1.20	0.59-2.32		Before-After	> 0.05 ns
Situation 4	After	1.17 (0.42)	1.10	0.68-2.12	0.004*	Before-After 1 year	< 0.05*
	After 1 year	1.53 (0.80)	1.31	0.78-3.92		After-After 1 year	< 0.05*
	Before	1.10 (0.46)	0.96	0.53-2.48		Before-After	
Situation 5	After	1.04 (0.45)	0.93	0.52-2.60	0.149	Before-After 1 year	> 0.05 ns
	After 1 year	1.25 (0.69)	1.08	0.55-3.68		After-After 1 year	
	Before	1.11 (0.39)	1.05	0.63-2.20		Before-After	
Situation 6	After	1.10 (0.52)	1.00	0.57-2.59	0.05 0	Before-After 1 year	> 0.05 ns
	After 1 year	1.31 (0.62)	1.22	0.54-3.50		After-After 1 year	
	Before	1.11 (0.43)	1.00	0.61-2.42		Before-After	
Situation 7	After	1.07 (0.49)	1.04	0.47-2.79	0.084	Before-After 1 year	> 0.05 ns
	After 1 year	1.27 (0.56)	1.18	0.53-2.63		After-After 1 year	
	Before	1.17 (0.46)	1.04	0.63-2.37		Before-After	> 0.05 ns
Situation 8	After	1.08 (0.53)	0.96	0.51-2.92	0.129	Before-After 1 year	< 0.05*
	After 1 year	1.28 (0.59)	1.09	0.70-3.21		After-After 1 year	< 0.05*
	Before	1.83 (0.89)	1.54	0.70-4.13		Before-After	> 0.05 ns
Situation 9	After	1.51 (0.63)	1.36	0.68-3.24	< 0.001*	Before-After 1 year	< 0.05*
	After 1 year	2.14 (0.90)	1.86	0.71-4.20		After-After 1 year	< 0.05*
	Before	1.91 (0.54)	1.87	0.78-2.80		Before-After	> 0.05 ns
Situation 10	After	1.62 (0.51)	1.47	0.85-2.76	0.008*	Before-After 1 year	< 0.05*
	After 1 year	2.10 (0.81)	1.95	1.00-4.19		After-After 1 year	< 0.05*

Friedman's non-parametric test. PC cm^2^ pressure center; Before: before the maneuver; After: after the maneuver; 12 months after the treatment; Situation 1: firm surface and open eyes; Situation 2: firm surface and closed eyes; Situation 3: pillow and eyes closed: Situation 4: firm surface and saccadic stimulation; Situation 5: firm surface and horizontal left/right optokinetic stimulation; Situation 6: firm surface and right/left horizontal optokinetic stimulation; Situation 7: firm surface and vertical top/bottom optokinetic stimulation; Situation 8: firm surface and bottom/up vertical optokinetic stimulation; Situation 9: firm surface and horizontal optokinetic stimulation associated with head movement; Situation 10: firm surface and vertical optokinetic stimulation associated with head movement; ns: not-significant.

There was a significant difference in BSV results in the following situations: 1 *(p=* 0.044), *2 (p=* 0.002), 3 *(p=* 0.001), 4 *(p=* 0.004), 9 *(p<* 0.001) and 10 *(p=* 0.008). In situations 1, 2, 3, and after treatment, the BSV differed significantly before *(p <* 0.05) and after 12 months of treatment *(p <* 0.05) and before treatment, BSV did not differ from that of 12 months after *(p >* 0.05) under the three situations mentioned. Under situations 4, 9 and 10 and 12 months after treatment, the BSV had a significant difference vis-à-vis the values obtained before *(p<* 0.05) and after *(p<* 0.05) treatment; however, the before treatment values were not different from those after treatment *(p>* 0.05). We also observed that there was no significant difference in BSV results among the three assessments under situations 5 *(p=* 0.149), *6(p=* 0.050), 7 *(p=* 0.084) and 8 *(p=* 0.129).

## DISCUSSION

Upon static posturography, the SL is established by the body movement the individual does on the support basis outlined by the feet, being able to utilize only the ankle to move. The SL found in the present study, 12 months after treatment for BPPV presented a value near that found in the study^11^, which assessed healthy elderly (Control Group) with ages higher than 65 years, and found SL values of 154.71 ± 55.10. One study carried out in 2009 compared the SL in patients diagnosed with BPPV, aged between 60 and 82 years; The SL mean value before the Epley maneuver was 134.27 and after the maneuver it was 181.03^12^.

We noticed that the SL values were not significantly different when the before, after and 12 months after treatment values were compared, which may be justified by the clinical characteristics of the BPPV, in which the body instability happens during intense fits, nonetheless, in a short duration, not interfering in the physical and functional aspects of the patient after the fit. However, one study carried out in 2009 reported a significant increase in the SL of elderly patients with BPPV when comparing the values before and after the Epley maneuver^12^. The difference found in both studies may be justified by the size of the sample assessed in each study. Although the difference was not significant, in the present study we found that the highest mean value found was immediately after the repositioning maneuver and that after 12 months without treatment there was SL reduction. Nonetheless, the mean value was higher than the one found before the repositioning maneuvers and in values similar to the ones found in healthy elderly (154 cm^2^)^11^.

When the PC area values in cm^2^ before, after and 12 months after treatment were compared, we noticed that in situations 1, 3, 4, 5, 6 and 7 there was no statistically significant difference in the three moments assessed. Situation 2 had a significant change in value, and the post-treatment moment was different from those before the maneuver and that 12 months after it. In such situation, there was a reduction in the PC cm^2^ area when we compared the pre and post maneuver moments, which increased again in value after 12 months of follow up. There was no significant difference between before and 12 months after treatment, indicating that after one year without follow up, the patient returns to having PC cm^2^ areas similar to those values presented before treatment for BPPV.

The mean values of PC cm^2^ areas obtained 12 months after treatment in situations 8, 9 and 10 were significantly higher when compared to the before and after maneuver moments. In situations 8 and 9, the before was not different from the after, nonetheless, there was a difference in situation 10. These values may suggest that such situations were worse after 12 months of treatment, for being the ones with the worst level of integration and responses from the sensory systems involved in the maintenance of body balance and that, with the increase in age in the sample, was worsen by dizziness recurrence, and may be directly impaired by aging. Some authors have mentioned that the statoconia from the utriculus macula, which alter the physiology of the semicircular canals, may change the sensitivity of its sensory receptors, causing permanent macular dysfunction^13^. The results from this study are partially different from the findings^14^, since the authors found significant differences in situations 2, 7, 8 and 9 before and after the repositioning maneuver, and in this study, as per aforementioned, differences were found only in situations 2 and 10. As with the present study, the authors did not report significant changes to the PC cm^2^ area in situation 1 (stable surface with eyes open), fostering studies which reported that individuals with labyrinth diseases use visual and somatosensory clues to control body balance^15^^,^^16^.

Values concerning BSV cm/s were significantly different in situations 1, 2 and 3 after treatment, when compared to before and 12 months after treatment. There was a reduction in BSV cm/s in situations 1, 2 and 3 when before values were compared to after, followed by increase after 12 months of treatment. We should stress that the lower the BSV values in cm/s, the better is the patient's body balance. Results from the mean values found in situations 1, 2 and 3 before and after the maneuver and 12 months afterwards, indicate the efficacy of the ORM after immediate treatment, nonetheless, 12 months afterwards without follow up, the patient returned to having body sways similar to those before treatment by the maneuvers. In one study, the authors reported the Epley maneuver's effectiveness, concerning the improvement in lateral body sway, nonetheless, there was no influence of the maneuver in the anteroposterior plane oscillation, which persisted for 12 months after treatment. They also suggest that the results indicated a change of unknown cause in the Spinal-Vestibular reflex (SVR)^17^.

In situations 4, 9 and 10, in which there were saccadic (4) and optokinetic stimuli, associated with head movement (9 and 10), there was a significant increase in BSV cm/s 12 months after treatment when compared to before and after treatment, thus showing an increase in visual-vestibular conflict, which may justify the increase in body sway in the situations aforementioned. According to Zee^18^, the oculovestibular reflex (OVR) degeneration is one of the factors associated with the vestibular system aging, thus it may suggest that the posturography sensory situations which assess visual responses associated with vestibular ones, and which depend on the proper working of the BSV, may be impaired in the elderly population. In a recent study published, in which they compared the results from a control group of elderly with those from a group of elderly submitted to the Epley's maneuver, it was seen that the individuals had PC cm^2^ and BSV cm/s values similar to the control group, ratifying the efficacy of the maneuver in the immediate treatment^19^.

Body balance recovery stems from the plasticity of the Central Nervous System (CNS)^20^. It is then suggested that this process is altered by aging associated with the BPPV, which would justify an increase in BSV cm/s in BRU^TM^ sensory situations, which require a greater skill from the OVR, even when compared to the pre-maneuver moment.

These signs after 12 months without follow up shows how fundamental is the long term follow up in the geriatric population with BPPV, considering that this population has a significant functional loss from one year to another, which may be increased by symptom recurrence.

## CONCLUSION

When the moments before and after the maneuver were compared, the static posturography showed a significant improvement in the body balance of the elderly population, showing ORM efficacy in the treatment of BPPV. However, after 12 months of treatment, the results showed changes in body balance similar to the moment before treatment.

Therefore, the results from this study enable the conclusion that static posturography, showing that there was an increase in body sway, pointing to a worse in body balance 12 months after treatment for BPPV.
